# Trends and outcomes of surgical treatment for ovarian cancer in older adults in Japan

**DOI:** 10.3389/fonc.2026.1743155

**Published:** 2026-05-04

**Authors:** Tsuyoshi Hisa, Michihide Maeda, Shinya Matsuzaki, Miho Kitai, Reisa Kakubari, Shoji Kamiura, Toshitaka Morishima, Isao Miyashiro, Seiji Mabuchi

**Affiliations:** 1 Department of Gynecology, Osaka International Cancer Institute, Osaka, Japan; 2 Department of Obstetrics and Gynecology, Osaka Keisatsu Hospital, Osaka, Japan; 3 Department of Obstetrics and Gynecology, The University of Osaka Hospital, Suita, Osaka, Japan; 4 Department of Obstetrics and Gynecology, Ashiya Municipal Hospital, Ashiya, Hyogo, Japan; 5 Cancer Control Center, Osaka International Cancer Institute, Osaka, Japan; 6 Department of Obstetrics and Gynecology, Hyogo Medical University, Nishinomiya, Hyogo, Japan

**Keywords:** aged, antineoplastic combined chemotherapy, cytoreduction surgical procedures, ovarian neoplasms, residual tumor, survival

## Abstract

**Objective:**

The prognostic implications of surgical outcomes in older adults with ovarian cancer remain unclarified. This study aimed to describe temporal trends in surgical management and examine the association between surgical outcomes and survival among older patients with ovarian cancer.

**Methods:**

This population-based retrospective study analyzed data from the Osaka Cancer Registry from patients diagnosed with ovarian cancer between 2004 and 2018. During this period, 10,033 women were diagnosed with malignant ovarian tumors in Osaka Prefecture, of whom 7,285 were classified as epithelial ovarian malignancies. Among them, 1,441 patients aged ≥70 years were identified. After excluding 39 patients with unknown surgical outcomes and 40 with insufficient chemotherapy data, 1,365 patients were included. Based on the surgical outcomes, patients were classified as having complete resection with no residual tumor, residual macroscopic tumor-present, or no surgery. Disease extent was categorized as localized, regional, or distant. Overall survival was assessed using univariate and multivariate models.

**Results:**

The proportion of patients managed without surgery increased over the study period, from 20.9% in 2004–2007 to 29.0% in 2016–2018, although this trend did not reach statistical significance (p = 0.11). This shift was most evident among patients with advanced disease (p < 0.001), indicating a temporal change in surgical management among older patients. A stepwise survival gradient was observed according to surgical outcome. The 5-year overall survival rates were **63.9%, 27.9%, and 9.9%** for complete resection, residual macroscopic tumor, and no surgery, respectively. Compared with complete resection, the hazard ratios for mortality were 2.63 (95% CI, 2.22–3.10) for residual macroscopic tumor and 5.37 (95% CI, 4.55–6.34) for no surgery. In multivariate analysis, surgical outcome remained associated with overall survival after adjustment for available clinical variables.

**Conclusion:**

In this registry-based cohort of patients aged ≥70 years, the proportion of patients managed without surgery increased over time, particularly among those with advanced disease, and receipt of surgery was associated with longer overall survival. However, as comorbidity and frailty data were unavailable in the registry, residual confounding related to treatment selection cannot be excluded.

## 1 Introduction

Ovarian cancer is one of the leading causes of cancer-related mortality in women, with a disproportionately higher mortality rate among older adults. In Japan, there are approximately 13,388 new cases of ovarian cancer diagnosed annually, and 4,876 deaths related to ovarian cancer annually. Among these cases of ovarian cancer in Japan, 8,337 are epithelial and sex-cord stromal malignancies, and 2,020 (25.4%) involve women aged 70 years and older (1).

The standard therapeutic strategy for ovarian cancer is primary debulking surgery followed by postoperative chemotherapy with platinum-based agents and taxanes. When primary surgical resection is not feasible because of advanced disease or poor clinical status, neoadjuvant chemotherapy (NACT) may be used. Advanced ovarian cancer is often treated with postoperative chemotherapy followed by maintenance therapy comprising anti-angiogenic agents or poly (ADP-ribose) polymerase (PARP) inhibitors.

The central aim of cytoreductive surgery (primary or interval debulking surgery) is complete macroscopic tumor resection. Postoperative residual disease is a well-established adverse prognostic factor (2–4), with worse outcomes in patients who forgo surgery than in those with residual tumors exceeding 1 cm (5). Moreover, outcomes in patients who do not undergo surgical resection following neoadjuvant chemotherapy have been reported to be comparable to those observed in patients with macroscopic residual disease (so-called R2 resection) (6), underscoring the prognostic relevance of achieving complete cytoreduction whenever feasible.

The negative impact of residual disease after surgery for ovarian cancer is particularly evident in older women (7), as older women undergo complete surgical resection less frequently and are less likely to receive guideline-concordant treatment (8–10). Among American patients aged 75 years and older with ovarian cancer in the Surveillance, Epidemiology, and End Results (SEER) database, only 37.6% underwent surgery, 51.2% received chemotherapy, and 18.9% received both surgery and chemotherapy (11). Similarly, an analysis of 28,165 women with ovarian cancer in the SEER database demonstrated that younger patients have a significantly higher likelihood of undergoing primary surgery than older patients (12). The treatment decisions in older women are often complicated by frailty and comorbidities; however, with appropriate performance status assessment and judicious selection, aggressive therapy can be feasible (13–16).

As life expectancy continues to rise, the number of older women with ovarian cancer is expected to increase (17). In Japan, women aged ≥70 years accounted for 28.4% of the population in 2019, and this proportion is projected to reach 35% by 2040 (18). Despite this demographic trend, older adults remain underrepresented in clinical trials, and population-based evidence describing treatment patterns and outcomes in this age group remains limited.

In the present study, we hypothesized that: (1) surgical treatment—particularly complete surgical resection—would be associated with improved overall survival (OS) among older adults; and (2) the proportion of patients managed without surgery has increased over time, especially among those with advanced-stage disease. To test these hypotheses, we analyzed data from the Osaka Cancer Registry (OCR) to evaluate temporal trends in surgical management and to examine the association between surgical outcomes and survival among elderly Japanese women—defined as those aged 70 years and older—diagnosed with epithelial ovarian cancer between 2004 and 2018. In Japan, individuals aged ≥65 years are generally defined as elderly, whereas those aged ≥75 years are categorized as late-stage elderly; therefore, the ≥70-year cutoff represents an intermediate threshold that has also been adopted in several prior studies of older patients with ovarian cancer. This population-based epidemiological analysis offers a comprehensive overview of real-world treatment patterns and clinical outcomes in a rapidly growing and historically underrepresented patient population.

## 2 Materials and methods

### 2.1 Data source

This retrospective observational study analyzed data from the population-based OCR. The OCR was established in 1962 and contains information on all malignancies diagnosed within Osaka Prefecture, Japan (19). Cancer cases are registered in the OCR through mandatory reporting from healthcare facilities and by linkage with death certificate databases. The OCR provides comprehensive demographic and clinical information, including patient sex, age at diagnosis, date of diagnosis, date of death, and the most recent follow-up findings regarding the vital status. Tumor-specific variables include the primary cancer site, histological classification, and extent of disease. Disease extent is categorized into three groups: 1) localized disease, defined as cancer confined to the organ of origin; 2) regional disease, involving regional lymph nodes and/or direct invasion of adjacent organs, including peritoneal dissemination; and 3) distant disease, indicating metastatic spread to remote organs. In accordance with the International Federation of Gynecology and Obstetrics (FIGO) 2014 staging system, localized disease corresponds with stage I, regional disease corresponds with stages II–III and IVA, and distant disease corresponds with stage IVB. Histological subtypes are determined using morphological codes from the International Classification of Diseases for Oncology (third edition). Treatment-related data includes the type of initial therapy, specifically surgery, chemotherapy, or radiotherapy. However, the OCR does not capture several important potential confounders, including socioeconomic status, comorbidity profiles, performance status, frailty indices, detailed subsequent treatments, or cause-specific mortality, all of which may influence both treatment selection and survival outcomes. Similarly, the registry lacks critical information regarding treatment details and sequencing. With respect to chemotherapy, the OCR does not provide data on specific regimens, dose intensity, treatment interruptions or modifications, or the use of targeted agents such as bevacizumab. Moreover, it is not possible to distinguish whether chemotherapy was administered as neoadjuvant therapy, adjuvant therapy, or treatment for recurrent disease. Information regarding treatment intent (palliative versus definitive) and overall treatment sequencing is also unavailable. Regarding surgery, the registry does not include detailed operative information, such as the extent of surgery (e.g., lymphadenectomy) or whether cytoreductive surgery was performed as primary debulking surgery or as interval debulking following neoadjuvant chemotherapy.

Follow-up to ascertain vital status is routinely conducted through linkage with national death certificate records (19).

### 2.2 Study population

Between January 2004 and December 2018, there were 10,033 women diagnosed with malignant ovarian tumors in Osaka Prefecture. Of these, 7,285 were classified as having epithelial ovarian malignancies. From this cohort, all patients aged 70 years and older were identified, yielding a total of 1,441 individuals. Following the exclusion of 39 patients with missing surgical outcome data and 40 with incomplete chemotherapy information, 1,365 patients were ultimately included in the present retrospective analysis.

Institutional Review Board approval was obtained prior to data extraction from the OCR. Variables collected comprised the age at diagnosis, extent of disease (localized [FIGO stage I], regional [FIGO stages II–III], distant [FIGO stage IV]), surgical treatment (performed or not), surgical outcomes (no residual tumor after cytoreductive surgery, residual macroscopic tumor after cytoreductive surgery, or no surgery), histologic subtype (serous carcinoma, endometrioid carcinoma, or other), administration of chemotherapy (yes or no), and diagnosis period (2004–2007, 2008–2011, 2012–2015, or 2016–2018). These factors were used for stratification in analyzes of overall survival (OS). The threshold of 70 years was selected as the age cutoff for consistency with prior studies (20, 21). In addition, we performed prespecified age-stratified analyzes using a cutoff of 80 years (≤79 vs. ≥80 years) to explore outcomes in the “oldest-old” group, which is clinically distinct and often managed differently in Japan; these analyzes are presented as subgroup (exploratory) analyzes. A sensitivity analysis was also performed excluding patients with a follow-up duration of less than 90 days to assess the potential impact of very early events on OS estimates.

### 2.3 Statistical analysis

Categorical variables were compared using the χ² test or Fisher’s exact test, as appropriate, while continuous variables were analyzed using the Mann–Whitney U test or the Kruskal–Wallis test, as applicable. Given the population-based design of this registry study, which included all eligible patients during the study period, a formal sample size or power calculation was not conducted. Therefore, the findings should be interpreted with appropriate caution, particularly in the context of multiple subgroup comparisons. Overall survival (OS) was estimated using the Kaplan–Meier method, with survival time defined as the interval from the date of diagnosis to death from any cause or last follow-up. Patients who were alive at the time of last follow-up were censored accordingly. Patients were categorized into three surgical groups: complete resection (no residual tumor), residual macroscopic tumor, and no surgery. The primary analysis evaluated overall survival across these three surgical groups. For comparison with previous studies, an additional two-group analysis (surgery vs. no surgery) was performed as a secondary analysis. Multivariate survival analyzes were performed using Cox proportional hazards regression models. All clinically relevant variables assessed in the univariate analyzes were entered simultaneously into the multivariate model, and no stepwise variable selection procedure was employed. All statistical tests were two-sided, and a p-value < 0.05 was considered statistically significant. Statistical analyzes were conducted using EZR software, version 1.27 (Saitama Medical Center, Jichi Medical University, Saitama, Japan) (22).

## 3 Results

### 3.1 Patients

The median follow-up duration was 33.6 months (range: 1.0–205.4 months). Of the 1,365 patients aged ≥70 years with epithelial ovarian cancer who were included in the final analysis, 339 (24.8%) were aged ≥80 years. We used the ≥80-year threshold for subgroup analyzes to evaluate outcomes among the oldest-old population.

Among the study cohort, 995 patients (72.9%) underwent surgery, comprising 615 (45.1%) with no residual disease and 380 (27.8%) with residual macroscopic disease. The remaining 370 patients (27.1%) did not undergo surgery. Histological subtype analysis was restricted to epithelial ovarian malignancies based on the Osaka Cancer Registry classification. The histologic subtypes were high-grade serous adenocarcinoma in 438 cases (32.1%), endometrioid adenocarcinoma in 149 (10.9%), mucinous adenocarcinoma in 185 (13.6%), clear cell adenocarcinoma in 164 (12.0%), and other subtypes in 429 (31.4%). The “other subtypes” category included unclassified adenocarcinoma and low-grade serous adenocarcinoma, as well as other less common epithelial histological subtypes.

The baseline characteristics and distribution of surgical outcomes in accordance with age, disease extent, histologic subtype, chemotherapy administration, and diagnosis period are summarized in **Table 1**. The no-surgery group more frequently had an age ≥80 years, presented with advanced disease, and had non-serous histology. In contrast, the residual macroscopic tumor-present group was characterized by greater proportions of advanced-stage disease, serous histology, and chemotherapy.

**Table 1:** Patient characteristics and correlations with surgical outcomes.

Variables	Total (n=1365)	No residual tumor (n=615)	Residual macroscopic tumor (n=380)	Op(−) (n=370)	P value
Age, years					
Median (IQR)	76 (72–79)	75 (72–78)	75 (72–78)	78 (74–82)	<0.001
70–79, n (%)	1026 (75.2%)	491 (79.8%)	309 (81.3%)	226 (61.1%)	
≥80, n (%)	339 (24.8%)	124 (20.2%)	71 (18.7%)	144 (38.9%)	
Disease extent, n (%)					<0.001
Local	281 (20.6%)	249 (40.5%)	17 (4.5%)	15 (4.1%)	
Regional	739 (54.1%)	309 (50.2%)	257 (67.6%)	173 (46.8%)	
Distant	345 (25.3%)	57 (9.3%)	106 (27.9%)	182 (49.1%)	
Histological type, n (%)					<0.001
Serous	438 (32.1%)	159 (25.9%)	185 (48.7%)	94 (25.4%)	
Endometrioid	149 (10.9%)	101 (16.4%)	38 (10.0%)	10 (2.7%)	
Others	778 (57.0%)	355 (57.7%)	157 (41.3%)	266 (71.9%)	
Chemotherapy, n (%)					<0.001
Yes	912 (66.8%)	366 (59.5%)	315 (82.9%)	231 (62.4%)	
No	453 (33.2%)	249 (40.5%)	65 (17.1%)	139 (37.6%)	
Diagnosis period, n (%)					0.02
2004–2007	230 (16.8%)	108 (17.6%)	74 (19.5%)	48 (13.0%)	
2008–2011	313 (22.9%)	124 (20.2%)	98 (25.8%)	91 (24.6%)	
2012–2015	439 (32.2%)	195 (31.7%)	124 (32.6%)	120 (32.4%)	
2016–2018	383 (28.1%)	188 (30.5%)	84 (22.1%)	111 (30.0%)	

IQR, interquartile range; Op(−), no surgery. Percentages are presented as column percentages.

### 3.2 Trends in surgical management

Temporal trends in surgical management are presented in **Table 2**. The proportion of patients in the no-surgery group increased from 20.9% during 2004–2007 to 29.0% during 2016–2018, although not statistically significant (p = 0.11). This rise was most evident among patients with advanced disease (*p* < 0.001); while 66% of patients with advanced disease underwent surgery in 2004–2007, most were managed without surgical intervention by 2012.

**Table 2:** Association between surgical outcomes and diagnosis period.

Group	Total (n=1365)	2004–2007 (n=230)	2008–2011 (n=313)	2012–2015 (n=439)	2016–2018 (n=383)	P value
A (All)						
Op (+), n (%)	995 (72.9%)	182 (79.1%)	222 (70.9%)	319 (72.7%)	272 (71.0%)	0.11
Op (−), n (%)	370 (27.1%)	48 (20.9%)	91 (29.1%)	120 (27.3%)	111 (29.0%)	
Total, n	1365 (100.0%)	230 (100.0%)	313 (100.0%)	439 (100.0%)	383 (100.0%)	
B (Local)						
Op (+), n (%)	266 (94.6%)	59 (95.2%)	54 (96.4%)	80 (95.2%)	73 (92.4%)	0.79
Op (−), n (%)	15 (5.4%)	3 (4.8%)	2 (3.6%)	4 (4.8%)	6 (7.6%)	
Total, n	281 (100.0%)	62 (100.0%)	56 (100.0%)	84 (100.0%)	79 (100.0%)	
C (Regional)						
Op (+), n (%)	566 (76.6%)	82 (77.4%)	125 (73.1%)	198 (79.8%)	161 (75.2%)	0.41
Op (−), n (%)	173 (23.4%)	24 (22.6%)	46 (26.9%)	50 (20.2%)	53 (24.8%)	
Total, n	739 (100.0%)	106 (100.0%)	171 (100.0%)	248 (100.0%)	214 (100.0%)	
D (Distant)						
Op (+), n (%)	163 (47.2%)	41 (66.1%)	43 (50.0%)	41 (38.3%)	38 (42.2%)	0.01
Op (−), n (%)	182 (52.8%)	21 (33.9%)	43 (50.0%)	66 (61.7%)	52 (57.8%)	
Total, n	345 (100.0%)	62 (100.0%)	86 (100.0%)	107 (100.0%)	90 (100.0%)	

Op(+), combined surgical group (no residual tumor and macroscopic residual tumor); Op(−), no surgery group; Percentages are presented as column percentages.

### 3.3 Association between surgical management and survival

**Figure 1:**
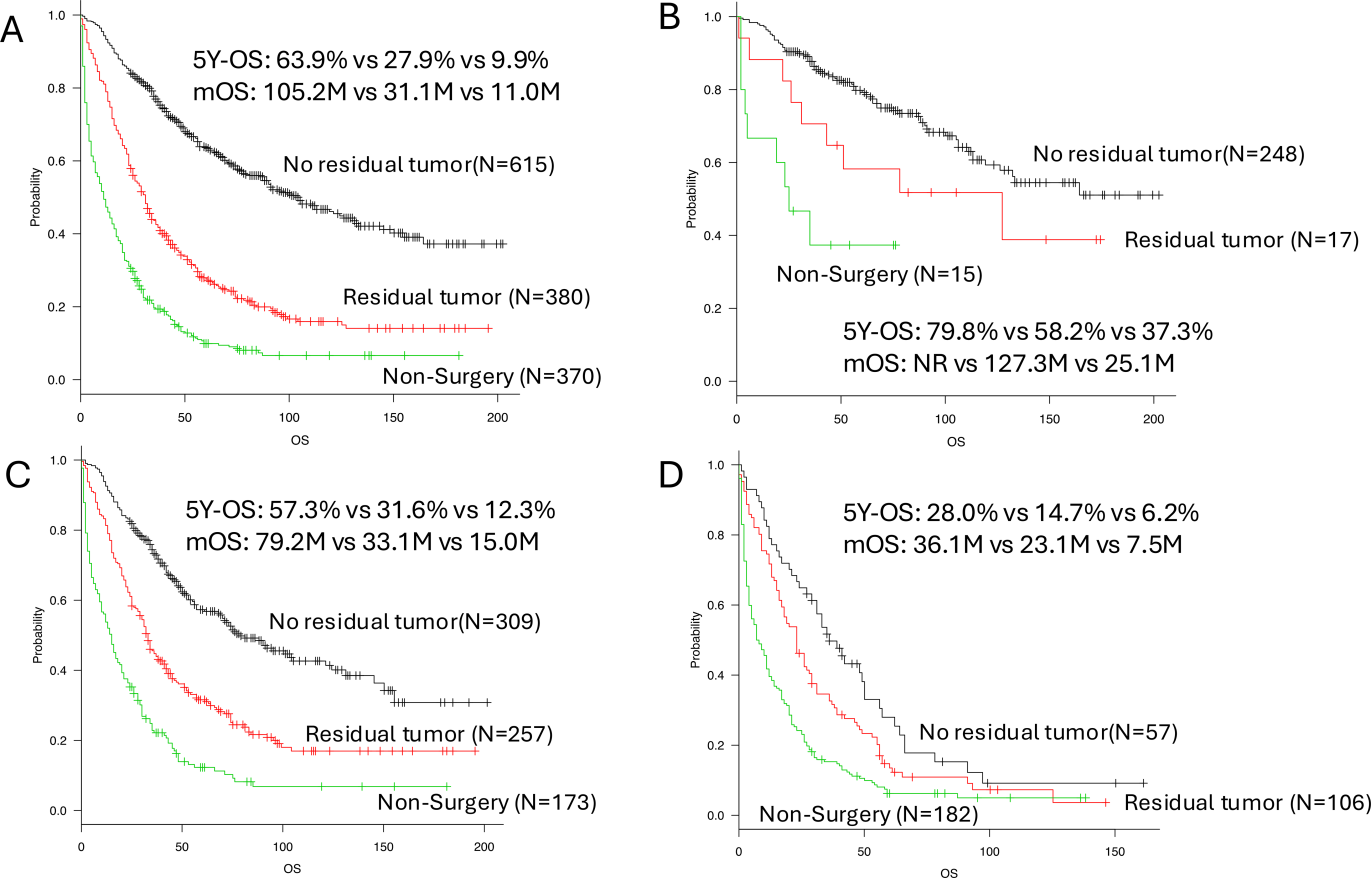
Comparison of OS, overall survival among surgical outcome groups: no surgery. Black line: no residual tumor; red line: residual tumor; green line: non-surgery. **(A)** All patients. **(B)** Patients with localized disease. **(C)** Patients with regional disease. **(D)** Patients with distant disease. mOS, median overall survival; HR, hazard ratio; CI, confidence interval.

The distribution of overall survival (OS) according to surgical status is shown in **Figure 1**. In univariate analysis (**Table 3**), a clear stepwise survival gradient was observed according to surgical outcome: patients who achieved complete resection (no residual tumor) had the most favorable prognosis, followed by those with residual macroscopic tumor, whereas patients who did not undergo surgery had the poorest survival.

**Table 3:** Univariate analysis of overall survival according to residual tumor status in patients aged ≥70 years.

Variable	All cases			Localized disease			Regional disease			Distant disease		
	n	HR (95% CI)	P value	n	HR (95% CI)	P value	n	HR (95% CI)	P value	n	HR (95% CI)	P value
Disease extent												
Local	281	1										
Regional	739	2.96 (2.37–3.70)	<0.001									
Distant	345	6.35 (5.01–8.04)	<0.001									
Surgical outcome												
No residual tumor	615	1		249	1		309	1		57	1	
Residual macroscopic tumor	380	2.63 (2.22- 3.10)	<0.001	17	1.77 (0.89- 3.55)	0.10	257	2.10 (1.69- 2.61)	<0.001	106	1.42 (0.99- 2.04)	0.05
Op (−)	370	5.37 (4.55- 6.34)	<0.001	15	5.23 (2.57- 10.64)	<0.001	173	4.21 (3.34- 5.30)	<0.001	182	2.43 (1.74- 3.40)	<0.001
Histology												
S + EM	587	1		92	1		367	1		128	1	
Others	778	1.18 (1.03–1.35)	0.02	189	1.01 (0.64–1.57)	0.98	372	1.14 (0.95–1.36)	0.16	217	1.95 (1.54–2.48)	<0.001
Chemotherapy												
Yes	912	1		95	1		570	1		247	1	
No	453	1.10 (0.95–1.27)	0.19	186	1.56 (0.99–2.48)	0.06	169	1.65 (1.35–2.02)	<0.001	98	2.82 (2.20–3.61)	<0.001
Diagnosis period												
2004–2007	230	1		62	1		106	1		62	1	
2008–2011	313	0.95 (0.79–1.15)	0.62	56	0.76 (0.46–1.27)	0.30	171	0.86 (0.66–1.13)	0.28	86	1.04 (0.75–1.46)	0.80
2012–2015	439	0.82 (0.68–0.99)	0.04	84	0.49 (0.28–0.86)	0.01	248	0.81 (0.62–1.05)	0.11	107	0.84 (0.61–1.16)	0.28
2016–2018	383	0.80 (0.65–0.98)	0.03	79	0.39 (0.18–0.83)	0.01	214	0.78 (0.58–1.04)	0.09	90	0.89 (0.63–1.25)	0.49

HR, hazard ratio; CI, confidence interval; n, number of patients; Op−, no surgery; S+EM, serous adenocarcinoma/endometrioid adenocarcinoma.

In the overall cohort, the 5-year OS rates were 63.9% for patients with no residual tumor, 27.9% for those with residual macroscopic tumor, and 9.9% for those who did not undergo surgery (**Figure 1A**). Compared with patients who achieved complete resection, the hazard ratios (HRs) for mortality were 2.63 (95% CI, 2.22–3.10; p < 0.001) for patients with residual macroscopic tumor and 5.37 (95% CI, 4.55–6.34; p < 0.001) for those who did not undergo surgery (**Table 3**). This stepwise association between surgical outcome and survival was consistently observed across disease-extent subgroups (**Figures 1B–D**). In multivariate analysis, surgical status remained independently associated with OS after adjustment for clinical variables (**Table 4**).

**Table 4:** Multivariate analysis of overall survival according to surgical treatment in patients aged ≥70 years, 70–79 years, and ≥80 years.

Variable	All cases (≥70 years)			70–79 years			≥80 years		
	n	HR (95% CI)	P value	n	HR (95% CI)	P value	n	HR (95% CI)	P value
Disease extent									
Local	281	1		219	1		62	1	
Regional	739	3.00 (2.35- 3.84)	<0.001	562	2.98 (2.20- 4.04)	<0.001	177	2.40 (1.52- 3.78)	<0.001
Distant	345	4.71 (3.59- 6.18)	<0.001	245	4.77 (3.42- 6.67)	<0.001	100	3.71 (2.25- 6.12)	<0.001
Surgical outcome									
No residual tumor	615	1		491	1		124	1	
Residual macroscopic tumor	380	2.14 (1.79- 2.56)	<0.001	309	2.09 (1.70- 2.57)	<0.001	71	2.12 (1.43- 3.12)	<0.001
Op (−)	370	3.81 (3.16- 4.59)	<0.001	226	3.34 (2.66- 4.21)	<0.001	144	3.93 (2.77- 5.57)	<0.001
Histology									
S + EM	587	1		466	1		121	1	
Others	778	1.11 (0.96- 1.28)	0.17	560	1.03 (0.85- 1.23)	0.79	218	1.33 (0.99- 1.80)	0.06
Chemotherapy									
Yes	912	1		775	1		137	1	
No	453	2.14 (1.83- 2.49)	<0.001	251	1.73 (1.40- 2.15)	<0.001	202	1.92 (1.46- 2.51)	<0.001
Diagnosis period									
2004–2007	230	1		185	1		45	1	
2008–2011	313	0.83 (0.68- 1.01)	0.07	240	0.79 (0.63- 1.00)	0.05	73	0.92 (0.62- 1.38)	0.70
2012–2015	439	0.76 (0.63- 0.92)	0.006	330	0.70 (0.56- 0.87)	0.002	109	0.88 (0.60- 1.31)	0.54
2016–2018	383	0.80 (0.64- 0.99)	0.04	271	0.71 (0.55- 0.93)	0.01	112	0.83 (0.55- 1.24)	0.36

HR, hazard ratio; CI, confidence interval; n, number of patients; Op (−), no-surgery group; S+EM, serous adenocarcinoma/endometrioid adenocarcinoma.

**Figure 2:**
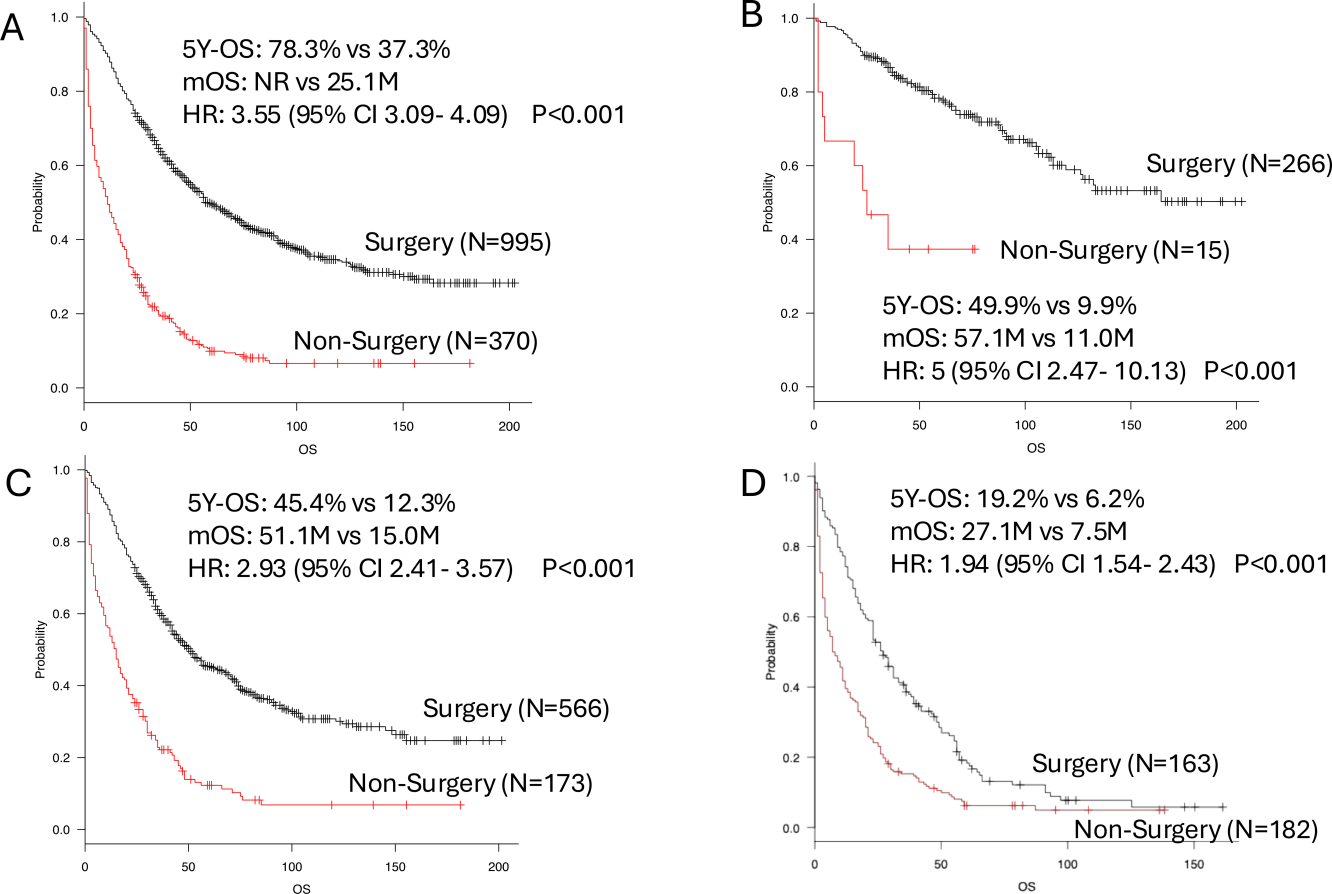
Kaplan–Meier curves of OS, overall survival in accordance with surgical intervention. Black line: surgery group; red line: non-surgery group. *P*-values are derived from log-rank tests. **(A)** All patients. **(B)** Patients with localized disease. **(C)** Patients with regional disease. **(D)** Patients with distant disese. mOS, median overall survival; HR, hazard ratio; CI, confidence interval.

To facilitate comparison with previous studies evaluating the potential impact of surgical intervention, we additionally examined survival outcomes using a two-group comparison (surgery vs. no surgery) (**Figure 2**). In this analysis, the 5-year OS rate was significantly lower in the no-surgery group than in the surgery group (37.3% vs. 78.3%; HR, 3.55; 95% CI, 3.09–4.09; p < 0.001), and this unfavorable prognosis was consistently observed across disease-extent subgroups (**Supplementary Tables S1**, **S2**).

**Figure 3:**
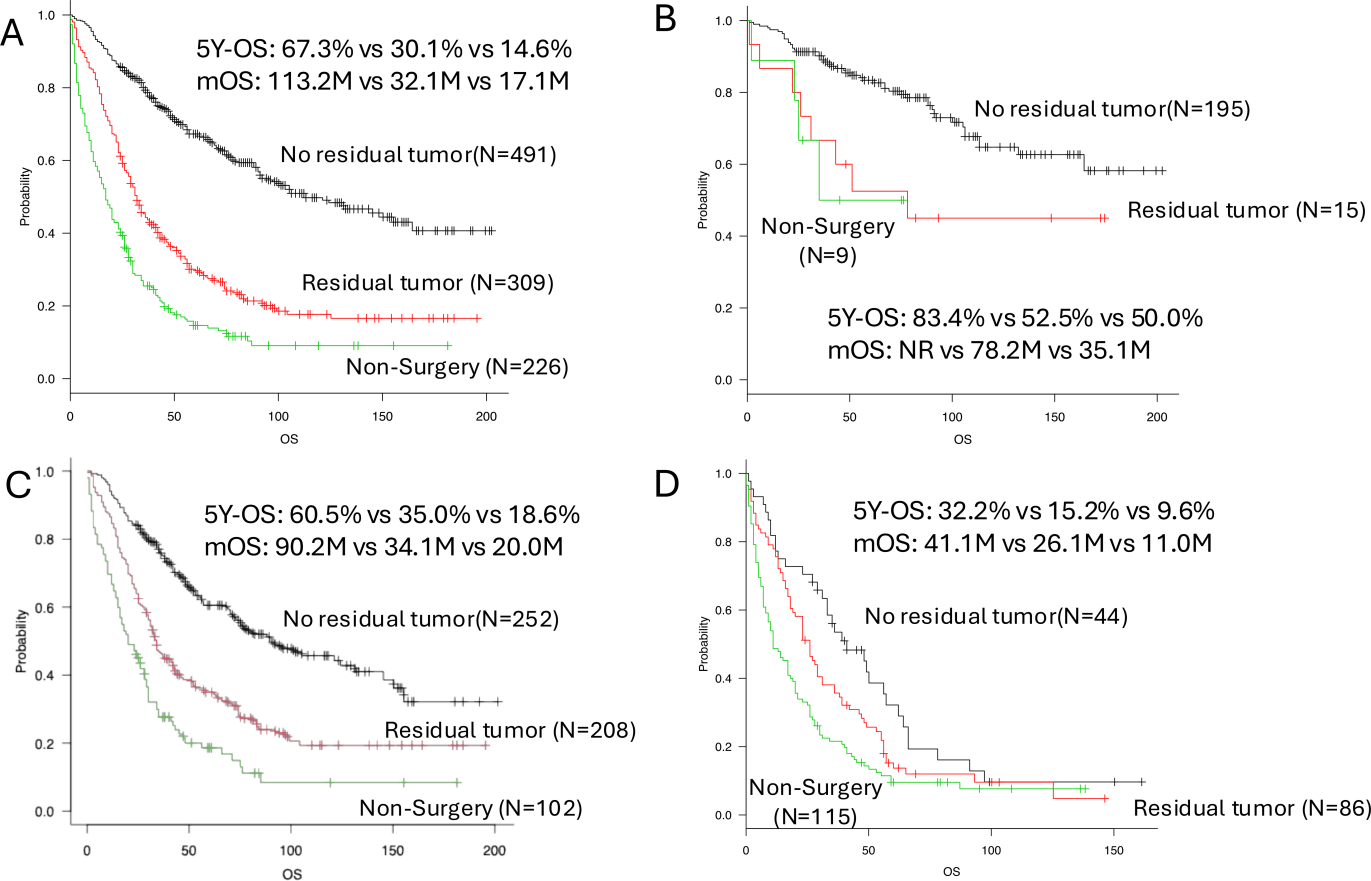
Comparison of OS, overall survival according to surgical outcome (no residual tumor versus residual tumor present) in patients aged 70–79 years. Black line: no residual tumor; red line: residual tumor; green line: non-surgery. **(A)**All cases. **(B)**Cases with local disease. **(C)** Cases with regional disease. **(D)** Cases with distant disease. mOS, Median overall survival; HR, Hazard ratio; CI, Confidence Interval; Residual tumor, residual macroscopic tumor present.

Among patients aged 70–79 years, the prognostic impact of surgical status is shown in **Figure 3**, **Supplementary Figure S1**. In this age group, the 5-year OS rates were 67.3% for patients with no residual tumor, 30.1% for those with residual macroscopic tumor, and 14.6% for those who did not undergo surgery (**Figure 3A**). Compared with complete resection, the HRs for mortality were 2.75 (95% CI, 2.27–3.32; p < 0.001) for residual tumor and 4.81 (95% CI, 3.92–5.89; p < 0.001) for no surgery (**Supplementary Table S3**). When surgical intervention was evaluated using a two-group comparison, the 5-year OS rate was significantly lower in the no-surgery group than in the surgery group (14.6% vs. 52.6%; HR, 3.09; 95% CI, 2.60–3.68; p < 0.001) (**Supplementary Figure S1A**). Disease-extent–specific survival curves for this age group are presented in **Figure 3**.

Among patients aged ≥80 years, survival outcomes are shown in **Supplementary Figures S2**, **S3**. In this age group, the 5-year OS rates were 49.9% for patients with no residual tumor, 18.0% for those with residual macroscopic tumor, and 2.4% for those who did not undergo surgery (**Supplementary Figure S2A**). Compared with complete resection, the HRs were 2.48 (95% CI, 1.74–3.52; p < 0.001) for residual tumor and 5.62 (95% CI, 4.14–7.64; p < 0.001) for no surgery (**Supplementary Table S4**). In the two-group comparison, the 5-year OS rate was significantly lower in the no-surgery group than in the surgery group (2.4% vs. 38.2%; HR, 3.95; 95% CI, 3.06–5.10; p < 0.001) (**Supplementary Figure S3**). Disease-extent–specific survival curves for this age group are shown in **Supplementary Figure S3A**.

The multivariate logistic regression analysis evaluating **factors associated with receipt of surgery** is summarized in **Table 5**. Significant predictors included age, histologic subtype, receipt of chemotherapy, disease stage, and year of diagnosis. Further stratification demonstrated that patients with three or more of these factors were more frequently managed without surgical intervention (**Supplementary Table S5**). To further evaluate the robustness of our findings, we performed additional analyzes. First, to assess the potential influence of very short follow-up, we repeated the analysis after excluding patients with a follow-up duration of less than 90 days (n = 109). The overall survival pattern remained unchanged. The 5-year OS rates were 64.6% for patients with no residual tumor, 29.2% for those with residual macroscopic tumor, and 13.0% for those who did not undergo surgery (**Supplementary Figure S4A**). Similar survival patterns across disease-extent subgroups are shown in **Supplementary Figures S4B–D**. Second, we conducted a subgroup analysis restricted to patients who received chemotherapy. In this cohort, the 5-year OS rates were 63.2% for patients with no residual tumor, 28.1% for those with residual macroscopic tumor, and 13.4% for those who did not undergo surgery (**Supplementary Figure S5A**). Comparable survival patterns across disease-extent categories are presented in **Supplementary Figures S5B–D**. These additional analyzes further support the robustness and consistency of the association between surgical outcome and overall survival.

**Table 5:** Multivariate analysis of factors influencing the decision to perform surgery.

Variable	Op (+) (n=995)		Op (−) (n=370)		OR (95% CI)	P value
	N	%	n	%		
Age						
<80	800	80.4	226	61.1	1	
≥80	195	19.6	144	38.9	2.15 (1.57–2.95)	<0.001
Histology						
S + EM	483	48.5	104	28.1	1	
Others	512	51.5	266	71.9	2.57 (1.93–3.43)	<0.001
Chemotherapy						
Yes	681	68.4	231	62.4	1	
No	314	31.6	139	37.6	1.51 (1.09–2.08)	0.01
Disease extent						
Local	266	26.7	15	4.0	1	
Regional	566	56.9	173	46.8	7.79 (4.38–13.90)	<0.001
Distant	163	16.4	182	49.2	27.50 (15.20–49.90)	<0.001
Diagnosis period						
2004–2011	404	40.6	139	37.6	1	
2012–2018	591	59.4	231	62.4	1.33 (1.00–1.76)	0.05

OR, odds ratio; CI, confidence interval; n, number of patients; Op (+), combined surgical group (no residual tumor and macroscopic residual tumor); Op (−), no-surgery group; S+EM, serous adenocarcinoma/endometrioid adenocarcinoma.

## 4 Discussion

While it is generally expected that poorer survival outcomes have been reported among patients who do not undergo surgery or who have residual disease after cytoreduction, our study provides population-based evidence in a rapidly aging Japanese population, demonstrating a stepwise survival gradient according to surgical outcome (complete resection, residual tumor, and no surgery), which persisted even among the oldest-old (≥80 years). Furthermore, we identified a temporal increase in the proportion of patients managed without surgery, particularly among those with distant disease. These findings may suggest a potential treatment gap in older adults and underscore the importance of careful patient selection when considering surgical management in older adults. To our knowledge, this study represents one of the largest population-based assessments of surgical treatment in older Japanese women with ovarian cancer.

In our cohort, serous histology accounted for 32% of cases, which appears relatively low compared with reports from Western countries. This likely reflects well-recognized ethnic and population-based differences in the histologic distribution of epithelial ovarian cancer. As reported by Machida et al, the histologic profile of ovarian cancer in Japan differs from that observed in Western countries (23). In their nationwide JSGO–JSOG collaborative study, serous carcinoma was the most common subtype (40.8%), followed by clear cell carcinoma (26.9%), endometrioid carcinoma (19.2%), and mucinous carcinoma (13.1%). In the present study, serous carcinoma comprised 32% of cases, which does not substantially deviate from these national data. Although the proportion of “other subtypes” was relatively high (approximately 40%) in our dataset, this category includes clear cell carcinoma—one of the most prevalent histologic subtypes in Japan. Accordingly, the comparatively lower proportion of serous carcinoma in our cohort should be interpreted within the context of established population-level differences in histologic patterns. Therefore, our findings should be understood within the framework of a Japanese population. It is conceivable that a similar population-based study conducted in Western countries—where serous and endometrioid carcinomas predominate—might yield somewhat different distributions and potentially different outcome patterns.

Demographic projections underscore the increasing clinical relevance of the present findings. In developed countries, the proportion of individuals aged ≥65 years was 17.5% in 2015 and is projected to rise to 27.4% by 2060. In Japan, this demographic transition is even more pronounced, with the proportion of older adults expected to reach approximately 35% by 2040 (18). As the population ages, the burden of ovarian cancer in older women will inevitably increase, further emphasizing the need for evidence-based treatment strategies tailored to this growing patient population.

Despite advances in surgical techniques, systemic therapy, and maintenance treatments, survival outcomes among older women with ovarian cancer remain suboptimal. Several factors may contribute to the observed age-related disparities in outcomes. First, older patients may present with more advanced-stage disease and potentially more aggressive tumor biology. Second, age-related changes in tumor characteristics or host physiology may be associated with reduced responsiveness to chemotherapy. Third, comorbidities, frailty, and nutritional deficiencies may increase treatment-related toxicity, limiting the feasibility of aggressive multimodal therapy. Finally, age-related bias in clinical decision-making may lead to less aggressive surgical management, suboptimal chemotherapy administration, and lower participation in clinical trials among older adults (24).

Although cytoreductive surgery can be performed in older adults with ovarian cancer, older patients frequently present with greater residual tumor burdens, which adversely affect both progression-free survival and OS (15, 16, 25, 26). One series of 280 women aged ≥65 years with advanced ovarian cancer showed that patients aged ≥80 years have a significantly higher prevalence of residual lesions >1 cm and a higher 3-month mortality rate than those aged 65–69 years (7). Similarly, Cloven et al. reported that an optimal surgical outcome is achieved in only 21.7% of women with ovarian surgery aged ≥80 years, compared with 29.5% of those aged 60–79 years and 73.7% of patients younger than 60 years (9). Gershenson et al. likewise demonstrated that the proportion of patients with ovarian cancer achieving optimal cytoreduction is lower in those aged ≥65 years compared with younger patients (33% vs. 61%) (27).

International data further highlight the undertreatment of older women with ovarian cancer. A nationwide cohort study of more than 23,000 patients with advanced ovarian cancer in the Netherlands reported that approximately one-third of older women receive no treatment at all (28). Similarly, a large retrospective analysis from France demonstrated that women aged ≥70 years with ovarian cancer are significantly less likely to undergo surgery or chemotherapy than younger patients, with only 31.9% of women aged ≥70 years receiving both modalities (20). Hightower et al. reported significantly worse survival in women aged ≥80 years with ovarian cancer compared with younger cohorts owing to less aggressive surgical management and reduced postoperative chemotherapy use (8). These age-related disparities in access to surgical intervention for ovarian cancer have been corroborated by multiple series (21, 29) and may be partly explained by lower referral rates to gynecologic oncologists among women aged ≥70 years (29). Consistent with previous reports, the proportions of patients aged ≥70 years in the present cohort who received no surgery and no chemotherapy were 27.1% and 33.2%, respectively. Among patients aged ≥80 years, the proportions of those who did not undergo surgery and did not receive chemotherapy were 57.5% and 40.4%, respectively.

According to a U.S. multicenter study, the likelihood of achieving complete cytoreduction in patients with ovarian cancer is influenced not only by tumor burden and distribution but also by patient resilience factors, including chronological age, performance status, nutritional status, and anesthetic risk (30). This previous study found that the groups at high risk of major morbidity and mortality are those older than 75 years, those with an American Society of Anesthesiologists score >3, those with a low preoperative serum albumin concentration, and those with an advanced disease stage (29). Despite the association between advanced age and surgical risk, several studies have reported that aggressive therapy for cancer can be tolerated in carefully selected older adult patients (13, 15, 17). For example, one study reported that an optimal surgical outcome was achieved in 74% of women aged ≥80 years with ovarian cancer, with acceptable postoperative complication rates (31). Multidisciplinary approaches also appear to improve outcomes. Earle et al. demonstrated that older women with ovarian cancer managed by gynecologic oncologists had a better prognosis compared with those treated by general gynecologists or surgeons (32). Furthermore, recent advances in geriatric assessment offer tools for more nuanced patient evaluation to potentially reduce undertreatment driven by incomplete clinical assessments (33). Importantly, when adjustments are made for FIGO stage, Eastern Cooperative Oncology Group performance status, and primary treatment type, age itself is no longer an independent predictor of adverse OS among patients with ovarian cancer (34). These previous findings are consistent with the present findings in highlighting the association between guideline-concordant treatment and improved outcomes, even in advanced age groups. However, because surgical exposure was analyzed from the time of diagnosis rather than from the actual timing of surgery, the observed association between surgery and survival may have been influenced by immortal-time (guarantee-time) bias. To address this concern, we conducted additional analyzes. First, a sensitivity analysis excluding patients with a follow-up duration of less than 90 days produced results consistent with the primary analysis, showing a similar stepwise survival gradient according to surgical outcome. Second, when the analysis was restricted to patients who received chemotherapy, the association between surgical outcome and overall survival remained similar. We believe that these additional analyzes indicate that the association between surgical outcome and survival did not materially change and was unlikely to be driven solely by very early deaths or differences in chemotherapy exposure.

We were unable to conduct an analysis comparing patients who underwent primary debulking surgery with those who received neoadjuvant chemotherapy followed by interval debulking surgery. However, neoadjuvant chemotherapy followed by interval debulking surgery has been shown to achieve comparable cytoreduction rates to primary surgery, with reduced intraoperative morbidity and mortality, making it a suitable alternative for frail patients (35). Longitudinal data from 9,587 women aged ≥70 years with stage II–IV ovarian cancer reveal a decrease in the use of primary surgery from 63.2% in 1991 to 49.5% in 2007, with a parallel significant increase in primary chemotherapy from 19.7% to 31.8%; however, OS rates remain largely unchanged (36). To improve the prognosis of older adults with ovarian cancer in Japan while maintaining their quality of life, it may be necessary to more effectively incorporate neoadjuvant chemotherapy and interval debulking surgery strategies.

### 4.1 Strengths

The principal strength of this study is its large-scale, population-based design using comprehensive registry data spanning 15 years. Unlike single-institution studies, this analysis reflects real-world treatment patterns in routine clinical practice. The inclusion of a substantial number of patients aged ≥80 years further enhances the clinical relevance of our findings in the context of Japan’s rapidly aging society.

### 4.2 Limitations

Several limitations of this study should be acknowledged. First, given the observational, registry-based design, selection bias is unavoidable. Patients who underwent surgery were likely fitter and more suitable candidates for aggressive treatment. Thus, the observed survival differences should be interpreted as associations rather than causal effects of surgery. Differences in baseline clinical condition, frailty, comorbidity burden, or treatment eligibility may have contributed to the observed survival disparities, and residual confounding cannot be excluded, as the apparent survival advantage associated with surgery may simply reflect the “healthy user effect,” comparing relatively robust patients who can tolerate surgery with frail patients who cannot. In addition, surgical exposure was analyzed from the time of diagnosis, whereas patients must survive long enough after diagnosis to undergo surgery in clinical practice. Because the registry does not record the date of surgery, the potential influence of immortal-time (or guarantee-time) bias cannot be excluded. Second, the registry lacks detailed treatment information. Changes in treatment practices during the study period (2004–2018), including increasing use of neoadjuvant chemotherapy (NACT) and the introduction of bevacizumab, could not be fully accounted for and may have influenced outcomes. Chemotherapy was recorded only as a binary variable (yes/no), without information on timing, regimens, dose intensity, cycle number, treatment completion, or targeted agents. Consequently, adjustment for chemotherapy exposure was crude and residual confounding may remain. In addition, it was not possible to determine whether chemotherapy was administered as neoadjuvant therapy without subsequent surgery, as definitive non-surgical treatment, for recurrent disease, or as palliative therapy. The registry also does not capture best supportive care. Therefore, poorer overall survival in the Op− group should not be interpreted as reflecting the complete absence of oncologic treatment, but rather the absence of surgical intervention within a heterogeneous treatment context. Third, cause-of-death data are not recorded in the OCR, precluding analysis of cancer-specific survival. Similarly, recurrence information is unavailable, preventing evaluation of progression-free survival. No predefined minimum follow-up period was applied; all available data were included up to each patient’s last observation, resulting in variable follow-up durations that may have influenced outcome assessment. Although some patients had very short follow-up due to early death or censoring, sensitivity analyzes excluding follow-up <90 days produced similar results (**Supplementary Figure S4**), suggesting minimal bias in OS estimates. Fourth, disease extent was classified using the SEER Summary Stage (localized, regional, distant), limiting more detailed evaluation of tumor burden, metastatic distribution, and FIGO substage–specific outcomes. Fifth, the study population was limited to Osaka Prefecture. Although highly urbanized and densely populated (approximately 9 million residents), Osaka represents only about 7.7% of Japan’s population, which may limit national generalizability. Moreover, it remains uncertain whether findings from a metropolitan region such as Osaka are applicable to other regions of Japan—particularly rural areas—or to countries with different healthcare systems, referral patterns, and demographic structures. Sixth, the impact of PARP inhibitors could not be assessed because they became available in Japan only in April 2018. Consequently, the study period largely predates widespread use of maintenance therapy, and future analyzes of more recent cohorts are needed to evaluate evolving treatment patterns and outcomes. Seventh, the Cox proportional hazards models assume proportional hazards over time. Given the long study period and evolving treatment strategies, this assumption may not have been fully satisfied; therefore, hazard ratios should be interpreted as average effects over the study period. Finally, no universally accepted definition of “older adult” exists in ovarian cancer research, with thresholds ranging from 60 to 80 years. We adopted ≥70 years for consistency with prior reports. In Japan, individuals aged ≥65 years are generally considered elderly, whereas those ≥75 years are categorized as late-stage elderly; thus, the ≥70-year cutoff represents a pragmatic compromise in the Japanese context. Nonetheless, alternative thresholds (e.g., ≥75 years) might yield different results, and our findings should be interpreted cautiously and validated in larger future studies.

## 5 Conclusions

This population-based analysis delineates treatment patterns and survival outcomes among older adults with ovarian cancer in Japan. Over the study period, the proportion of patients managed without surgery increased, particularly among those with advanced disease, indicating a temporal shift in surgical management in this older population. Patients who underwent surgery—particularly those achieving no residual tumor—had longer overall survival than those who did not undergo surgery; however, this finding represents an observed association within a population-based cohort rather than a causal effect. These results underscore the importance of careful patient selection when considering surgical management in older adults. Nevertheless, these findings should be interpreted cautiously given the observational design, the potential for residual confounding, and the multiple subgroup analyzes performed. Future research should further evaluate the feasibility and potential role of cytoreductive surgery and neoadjuvant chemotherapy followed by interval debulking surgery in carefully selected older patients. More comprehensive and detailed registry data are needed to enable refined analyzes and to support evidence-based clinical decision-making for this rapidly growing patient population.

## Data Availability

The data that support the findings of this study are available from the corresponding author upon reasonable request.
