# Supplementary material for: Trends and outcomes of surgical treatment for ovarian cancer in older adults in Japan
Source: Front Oncol. 2026 May 4;16:1743155. doi: 10.3389/fonc.2026.1743155 (PMC13180912; doi:10.3389/fonc.2026.1743155)
Supplement: Supplementary file 1 [file Presentation1.pptx]

## Slide 1
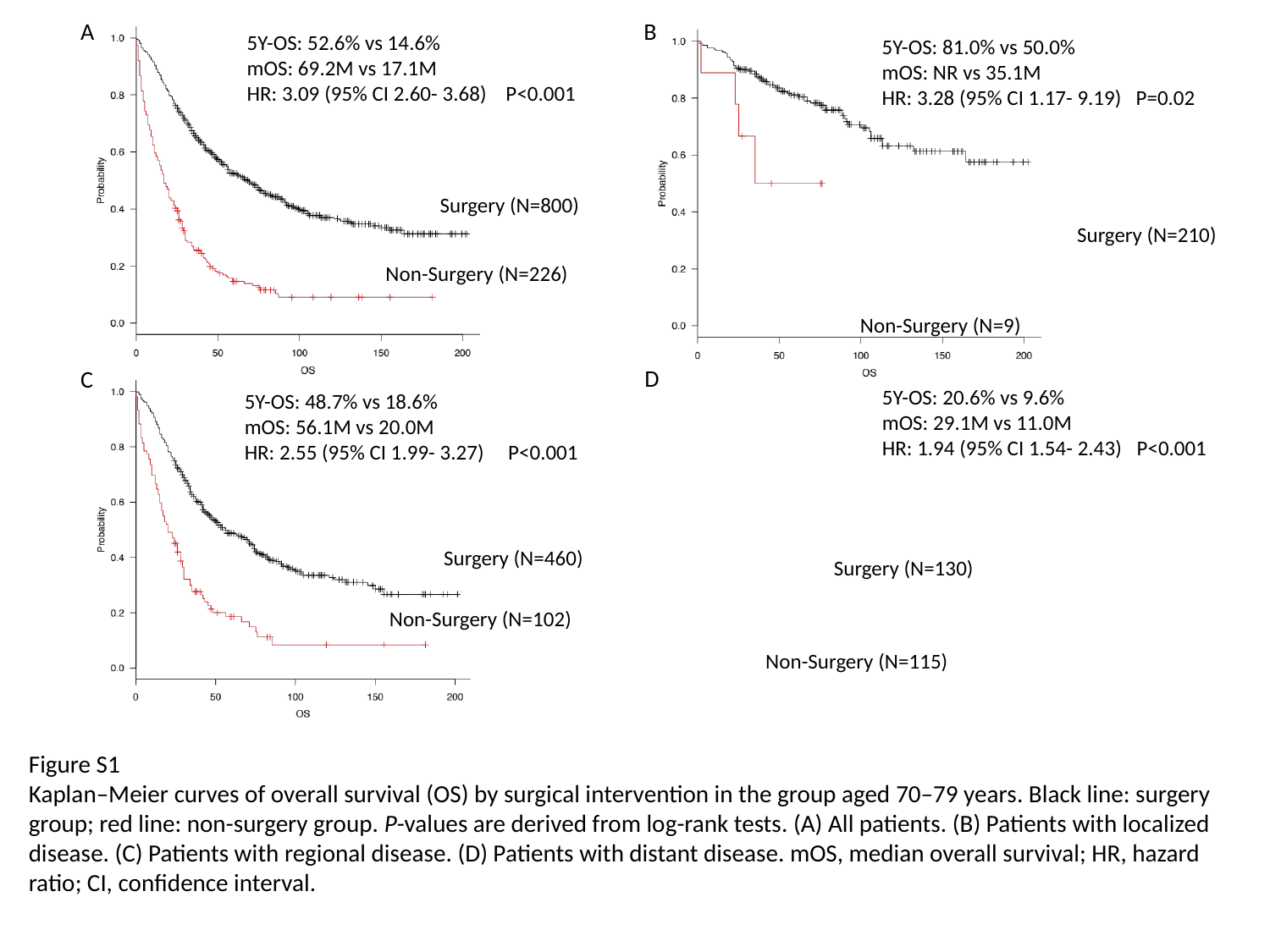

A
B
5Y-OS: 52.6% vs 14.6%
mOS: 69.2M vs 17.1M
HR: 3.09 (95% CI 2.60- 3.68) P<0.001
5Y-OS: 81.0% vs 50.0%
mOS: NR vs 35.1M
HR: 3.28 (95% CI 1.17- 9.19) P=0.02
Surgery (N=800)
Surgery (N=210)
Non-Surgery (N=226)
Non-Surgery (N=9)
D
C
5Y-OS: 20.6% vs 9.6%
mOS: 29.1M vs 11.0M
HR: 1.94 (95% CI 1.54- 2.43) P<0.001
5Y-OS: 48.7% vs 18.6%
mOS: 56.1M vs 20.0M
HR: 2.55 (95% CI 1.99- 3.27) P<0.001
Surgery (N=460)
Surgery (N=130)
Non-Surgery (N=102)
Non-Surgery (N=115)
Figure S1
Kaplan–Meier curves of overall survival (OS) by surgical intervention in the group aged 70–79 years. Black line: surgery group; red line: non-surgery group. P-values are derived from log-rank tests. (A) All patients. (B) Patients with localized disease. (C) Patients with regional disease. (D) Patients with distant disease. mOS, median overall survival; HR, hazard ratio; CI, confidence interval.

## Slide 2
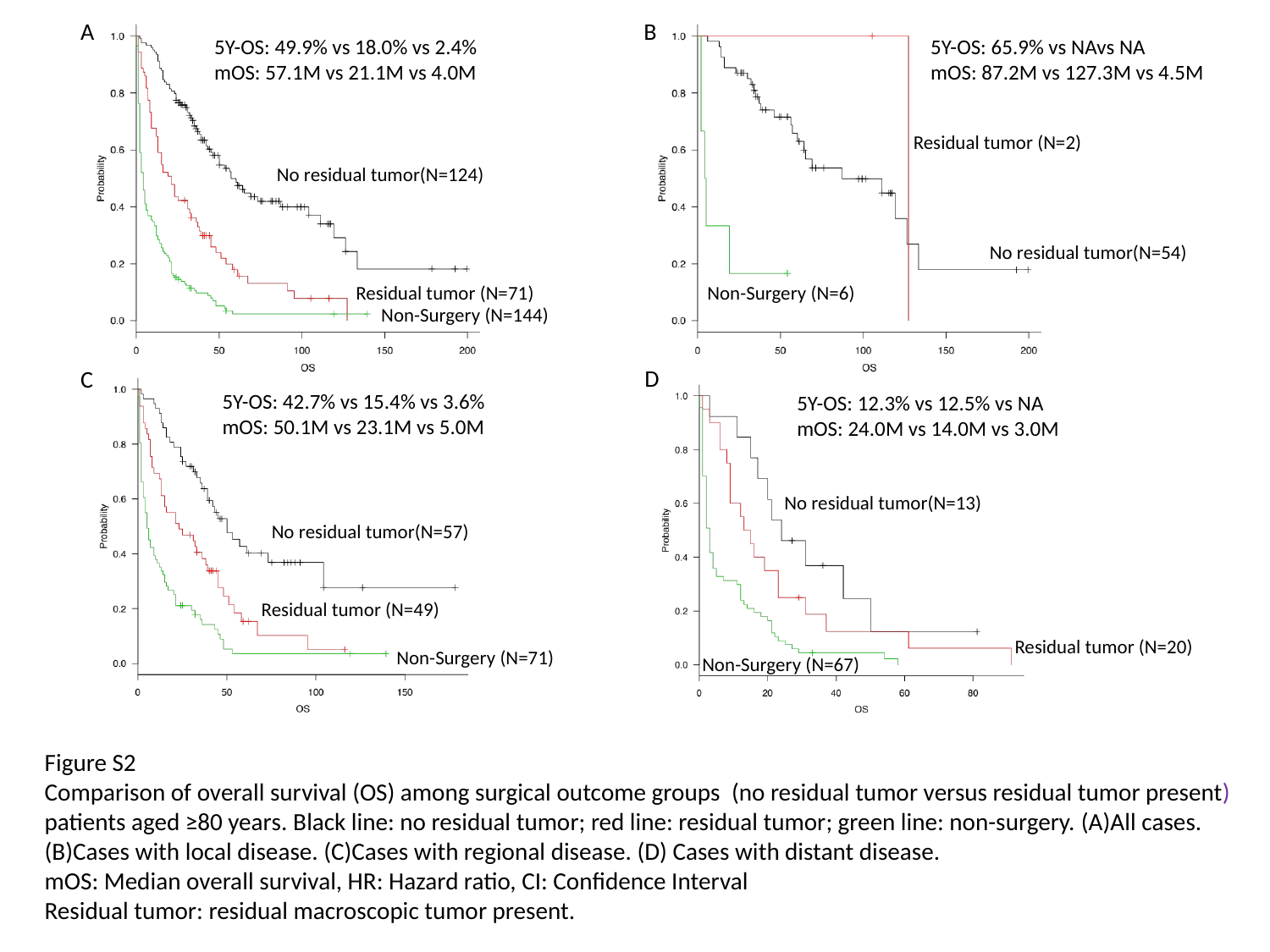

A
B
5Y-OS: 65.9% vs NAvs NA
mOS: 87.2M vs 127.3M vs 4.5M
5Y-OS: 49.9% vs 18.0% vs 2.4%
mOS: 57.1M vs 21.1M vs 4.0M
Residual tumor (N=2)
No residual tumor(N=124)
No residual tumor(N=54)
Non-Surgery (N=6)
Residual tumor (N=71)
Non-Surgery (N=144)
D
C
5Y-OS: 42.7% vs 15.4% vs 3.6%
mOS: 50.1M vs 23.1M vs 5.0M
5Y-OS: 12.3% vs 12.5% vs NA
mOS: 24.0M vs 14.0M vs 3.0M
No residual tumor(N=13)
No residual tumor(N=57)
Residual tumor (N=49)
Residual tumor (N=20)
Non-Surgery (N=71)
Non-Surgery (N=67)
Figure S2
Comparison of overall survival (OS) among surgical outcome groups (no residual tumor versus residual tumor present) patients aged ≥80 years. Black line: no residual tumor; red line: residual tumor; green line: non-surgery. (A)All cases. (B)Cases with local disease. (C)Cases with regional disease. (D) Cases with distant disease.
mOS: Median overall survival, HR: Hazard ratio, CI: Confidence Interval
Residual tumor: residual macroscopic tumor present.

## Slide 3
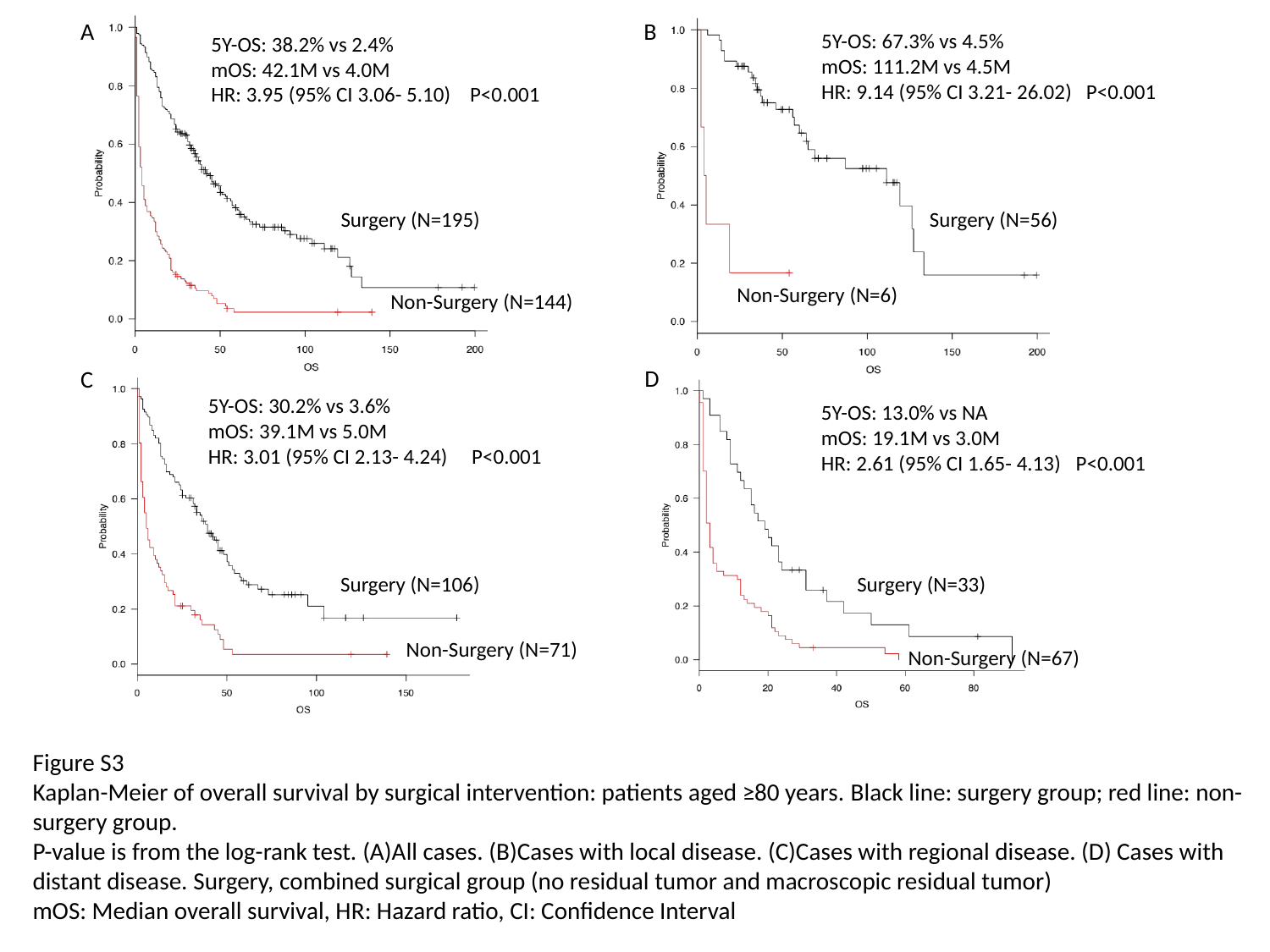

A
B
5Y-OS: 67.3% vs 4.5%
mOS: 111.2M vs 4.5M
HR: 9.14 (95% CI 3.21- 26.02) P<0.001
5Y-OS: 38.2% vs 2.4%
mOS: 42.1M vs 4.0M
HR: 3.95 (95% CI 3.06- 5.10) P<0.001
Surgery (N=195)
Surgery (N=56)
Non-Surgery (N=6)
Non-Surgery (N=144)
D
C
5Y-OS: 30.2% vs 3.6%
mOS: 39.1M vs 5.0M
HR: 3.01 (95% CI 2.13- 4.24) P<0.001
5Y-OS: 13.0% vs NA
mOS: 19.1M vs 3.0M
HR: 2.61 (95% CI 1.65- 4.13) P<0.001
Surgery (N=106)
Surgery (N=33)
Non-Surgery (N=71)
Non-Surgery (N=67)
Figure S3
Kaplan-Meier of overall survival by surgical intervention: patients aged ≥80 years. Black line: surgery group; red line: non-surgery group.
P-value is from the log-rank test. (A)All cases. (B)Cases with local disease. (C)Cases with regional disease. (D) Cases with distant disease. Surgery, combined surgical group (no residual tumor and macroscopic residual tumor)
mOS: Median overall survival, HR: Hazard ratio, CI: Confidence Interval

## Slide 4
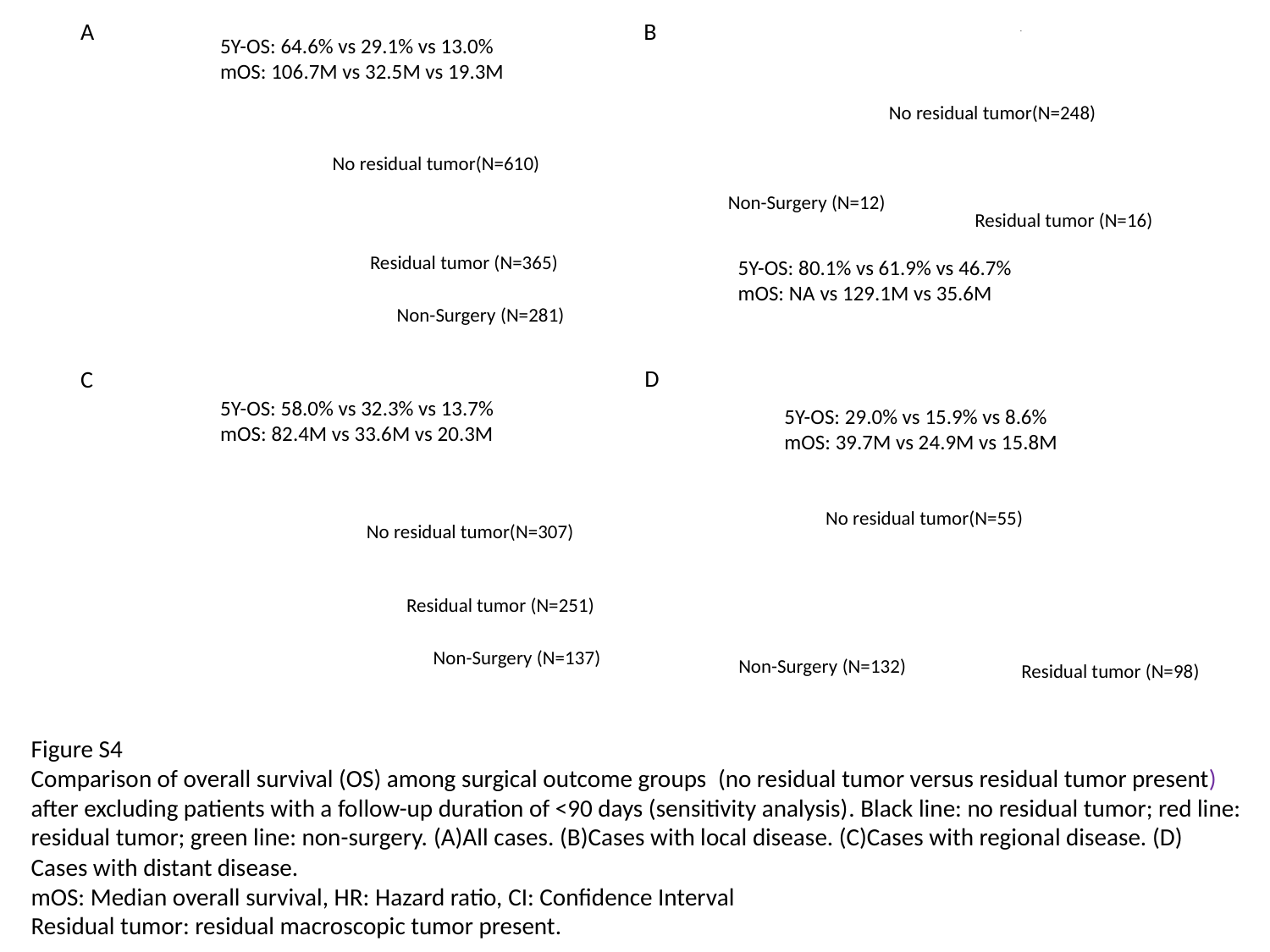

A
B
5Y-OS: 64.6% vs 29.1% vs 13.0%
mOS: 106.7M vs 32.5M vs 19.3M
No residual tumor(N=248)
No residual tumor(N=610)
Non-Surgery (N=12)
Residual tumor (N=16)
Residual tumor (N=365)
5Y-OS: 80.1% vs 61.9% vs 46.7%
mOS: NA vs 129.1M vs 35.6M
Non-Surgery (N=281)
D
C
5Y-OS: 58.0% vs 32.3% vs 13.7%
mOS: 82.4M vs 33.6M vs 20.3M
5Y-OS: 29.0% vs 15.9% vs 8.6%
mOS: 39.7M vs 24.9M vs 15.8M
No residual tumor(N=55)
No residual tumor(N=307)
Residual tumor (N=251)
Non-Surgery (N=137)
Residual tumor (N=98)
Non-Surgery (N=132)
Figure S4
Comparison of overall survival (OS) among surgical outcome groups (no residual tumor versus residual tumor present) after excluding patients with a follow-up duration of <90 days (sensitivity analysis). Black line: no residual tumor; red line: residual tumor; green line: non-surgery. (A)All cases. (B)Cases with local disease. (C)Cases with regional disease. (D) Cases with distant disease.
mOS: Median overall survival, HR: Hazard ratio, CI: Confidence Interval
Residual tumor: residual macroscopic tumor present.

## Slide 5
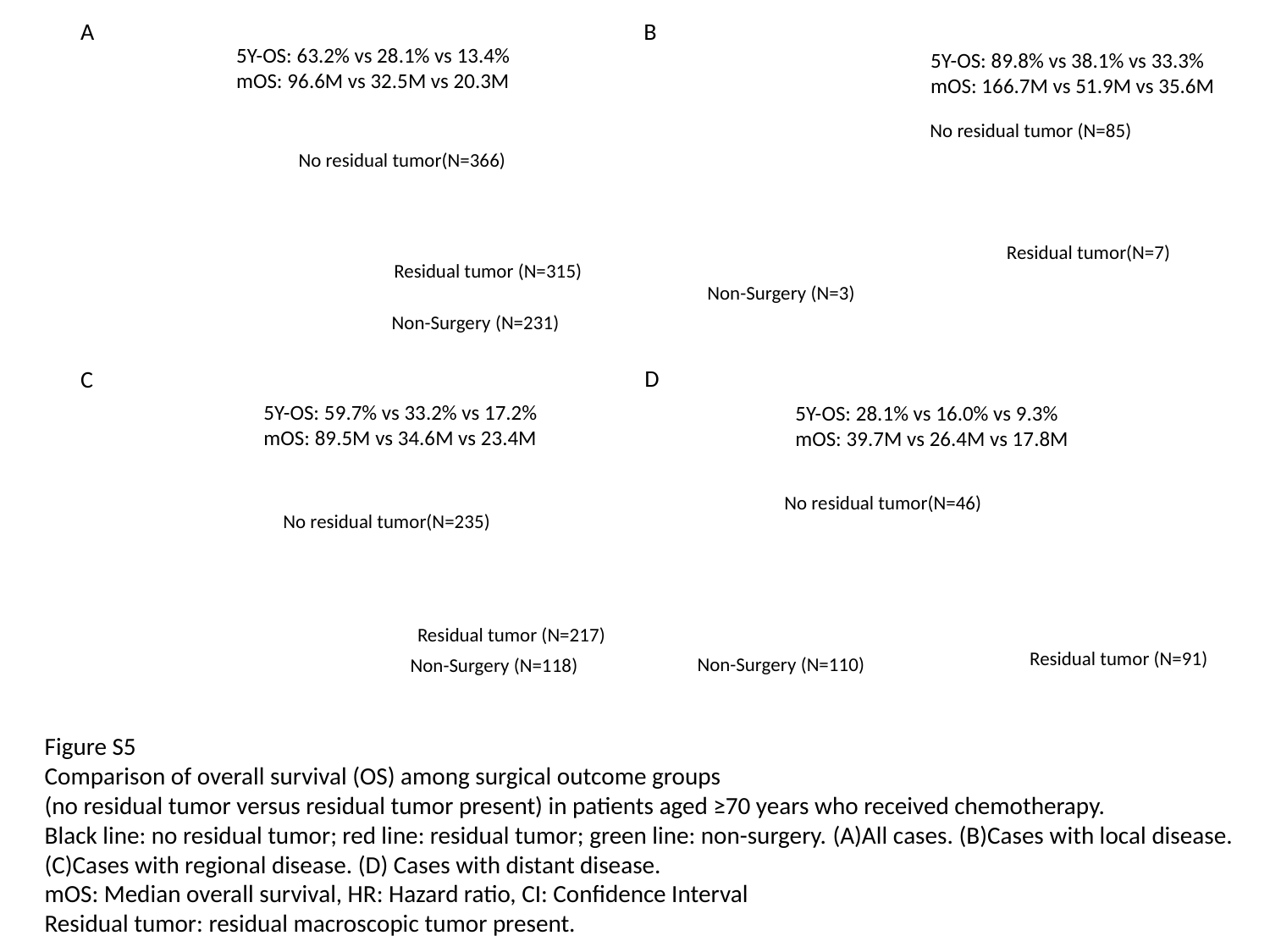

A
B
5Y-OS: 63.2% vs 28.1% vs 13.4%
mOS: 96.6M vs 32.5M vs 20.3M
5Y-OS: 89.8% vs 38.1% vs 33.3%
mOS: 166.7M vs 51.9M vs 35.6M
No residual tumor (N=85)
No residual tumor(N=366)
Residual tumor(N=7)
Residual tumor (N=315)
Non-Surgery (N=3)
Non-Surgery (N=231)
D
C
5Y-OS: 59.7% vs 33.2% vs 17.2%
mOS: 89.5M vs 34.6M vs 23.4M
5Y-OS: 28.1% vs 16.0% vs 9.3%
mOS: 39.7M vs 26.4M vs 17.8M
No residual tumor(N=46)
No residual tumor(N=235)
Residual tumor (N=217)
Residual tumor (N=91)
Non-Surgery (N=110)
Non-Surgery (N=118)
Figure S5
Comparison of overall survival (OS) among surgical outcome groups (no residual tumor versus residual tumor present) in patients aged ≥70 years who received chemotherapy.
Black line: no residual tumor; red line: residual tumor; green line: non-surgery. (A)All cases. (B)Cases with local disease. (C)Cases with regional disease. (D) Cases with distant disease.
mOS: Median overall survival, HR: Hazard ratio, CI: Confidence Interval
Residual tumor: residual macroscopic tumor present.
