# Supplementary material for: Trends and outcomes of surgical treatment for ovarian cancer in older adults in Japan
Source: Front Oncol. 2026 May 4;16:1743155. doi: 10.3389/fonc.2026.1743155 (PMC13180912; doi:10.3389/fonc.2026.1743155)
Supplement: Supplementary file 2 [file Table1.docx]

Table S1. Univariate analysis of factors associated with overall survival in patients aged ≥70 years.

| **Variable** | **All cases** | | | **Localized disease** | | | **Regional disease** | | | **Distant disease** | | |
| --- | --- | --- | --- | --- | --- | --- | --- | --- | --- | --- | --- | --- |
|  | n | HR (95% CI) | P value | n | HR (95% CI) | P value | n | HR (95% CI) | P value | n | HR (95% CI) | P value |
| **Disease extent** |  |  |  |  |  |  |  |  |  |  |  |  |
| Local | 281 | 1 |  |  |  |  |  |  |  |  |  |  |
| Regional | 739 | 2.96 (2.37–3.70) | <0.001 |  |  |  |  |  |  |  |  |  |
| Distant | 345 | 6.35 (5.01–8.04) | <0.001 |  |  |  |  |  |  |  |  |  |
| **Surgery** |  |  |  |  |  |  |  |  |  |  |  |  |
| Op (+) | 995 | 1 |  | 266 | 1 |  | 566 | 1 |  | 163 | 1 |  |
| Op (−) | 370 | 3.55 (3.09–4.09) | <0.001 | 15 | 5 (2.47–10.13) | <0.001 | 173 | 2.93 (2.41–3.57) | <0.001 | 182 | 1.94 (1.54–2.43) | <0.001 |
| **Histology** |  |  |  |  |  |  |  |  |  |  |  |  |
| S + EM | 587 | 1 |  | 92 | 1 |  | 367 | 1 |  | 128 | 1 |  |
| Others | 778 | 1.18 (1.03–1.35) | 0.02 | 189 | 1.01 (0.64–1.57) | 0.98 | 372 | 1.14 (0.95–1.36) | 0.16 | 217 | 1.95 (1.54–2.48) | <0.001 |
| **Chemotherapy** |  |  |  |  |  |  |  |  |  |  |  |  |
| Yes | 912 | 1 |  | 95 | 1 |  | 570 | 1 |  | 247 | 1 |  |
| No | 453 | 1.10 (0.95–1.27) | 0.19 | 186 | 1.56 (0.99–2.48) | 0.06 | 169 | 1.65 (1.35–2.02) | <0.001 | 98 | 2.82 (2.20–3.61) | <0.001 |
| **Diagnosis period** |  |  |  |  |  |  |  |  |  |  |  |  |
| 2004–2007 | 230 | 1 |  | 62 | 1 |  | 106 | 1 |  | 62 | 1 |  |
| 2008–2011 | 313 | 0.95 (0.79–1.15) | 0.62 | 56 | 0.76 (0.46–1.27) | 0.30 | 171 | 0.86 (0.66–1.13) | 0.28 | 86 | 1.04 (0.75–1.46) | 0.80 |
| 2012–2015 | 439 | 0.82 (0.68–0.99) | 0.04 | 84 | 0.49 (0.28–0.86) | 0.01 | 248 | 0.81 (0.62–1.05) | 0.11 | 107 | 0.84 (0.61–1.16) | 0.28 |
| 2016–2018 | 383 | 0.80 (0.65–0.98) | 0.03 | 79 | 0.39 (0.18–0.83) | 0.01 | 214 | 0.78 (0.58–1.04) | 0.09 | 90 | 0.89 (0.63–1.25) | 0.49 |

HR, hazard ratio; CI, confidence interval; n, number of patients; Op (+), combined surgical group (no residual tumor and macroscopic residual tumor); Op (−), no-surgery group; S+EM, serous adenocarcinoma/endometrioid adenocarcinoma.

Table S2. Multivariate analysis of factors associated with overall survival in patients aged ≥70 years.

| **Variable** | **All cases** | | | **70–79 years** | | | **≥80 years** | | |
| --- | --- | --- | --- | --- | --- | --- | --- | --- | --- |
|  | n | HR (95% CI) | P value | n | HR (95% CI) | P value | n | HR (95% CI) | P value |
| **Disease extent** |  |  |  |  |  |  |  |  |  |
| Local | 281 | 1 |  | 219 | 1 |  | 62 | 1 |  |
| Regional | 739 | 3.73 (2.94–4.73) | <0.001 | 562 | 3.66 (2.73- 4.91) | <0.001 | 177 | 3.02 (1.96- 4.65) | <0.001 |
| Distant | 345 | 6.28 (4.84–8.16) | <0.001 | 245 | 6.33 (4.60- 8.71) | <0.001 | 100 | 4.97 (3.10- 7.97) | <0.001 |
| **Surgery** |  |  |  |  |  |  |  |  |  |
| Op (+) | 995 | 1 |  | 800 | 1 |  | 195 | 1 |  |
| Op (−) | 370 | 2.56 (2.20–2.99) | <0.001 | 226 | 2.22 (1.84- 2.69) | <0.001 | 144 | 2.76 (2.09- 3.65) | <0.001 |
| **Histology** |  |  |  |  |  |  |  |  |  |
| S + EM | 587 | 1 |  | 466 | 1 |  | 121 | 1 |  |
| Others | 778 | 1.06 (0.92–1.22) | 0.42 | 560 | 1.00 (0.84- 1.18) | 0.95 | 218 | 1.25 (0.95- 1.65) | 0.11 |
| **Chemotherapy** |  |  |  |  |  |  |  |  |  |
| Yes | 912 | 1 |  | 775 | 1 |  | 137 | 1 |  |
| No | 453 | 1.97 (1.70–2.30) | <0.001 | 251 | 1.55 (1.25- 1.92) | <0.001 | 202 | 1.86 (1.43- 2.44) | <0.001 |
| **Diagnosis period** |  |  |  |  |  |  |  |  |  |
| 2004–2007 | 230 | 1 |  | 185 | 1 |  | 45 | 1 |  |
| 2008–2011 | 313 | 0.80 (0.65–0.97) | 0.02 | 240 | 0.77 (0.62- 0.97) | 0.02 | 73 | 0.85 (0.57- 1.27) | 0.43 |
| 2012–2015 | 439 | 0.71 (0.59–0.86) | <0.001 | 330 | 0.67 (0.54- 0.84) | <0.001 | 109 | 0.74 (0.50- 1.08) | 0.12 |
| 2016–2018 | 383 | 0.73 (0.59–0.90) | 0.003 | 271 | 0.67 (0.51- 0.86) | 0.002 | 112 | 0.72 (0.48- 1.07) | 0.11 |

HR, hazard ratio; CI, confidence interval; n, number of patients; Op (+),combined surgical group (no residual tumor and macroscopic residual tumor); Op (−), no-surgery group; S+EM, serous adenocarcinoma/endometrioid adenocarcinoma.

Table S3. Univariate analysis of overall survival according to surgical outcome in patients aged 70–79 years.

|  | **All cases** | | | **Local disease** | | | **Regional disease** | | | **Distant disease** | | |
| --- | --- | --- | --- | --- | --- | --- | --- | --- | --- | --- | --- | --- |
|  | n | HR (95% CI) | P value | n | HR (95% CI) | P value | n | HR (95% CI) | P value | n | HR (95% CI) | P value |
| Disease extent  Local  Regional  Distant | 219  562  245 | 1  3.19 (2.43- 4.19)  6.83 (5.12- 9.11) | <0.001  <0.001 |  |  |  |  |  |  |  |  |  |
| Surgical outcome  No residual tumor  Residual macroscopic tumor  Op (−) | 491  309  226 | 1  2.75 (2.27- 3.32)  4.81 (3.92- 5.89) | <0.001  <0.001 | 195  15  9 | 1  2.41 (1.14- 5.10)  3.57 (1.27- 10.08) | 0.02  0.02 | 252  208  102 | 1  2.11 (1.66- 2.70)  3.68 (2.76- 4.89) | <0.001  <0.001 | 44  86  115 | 1  1.42 (0.94- 2.12)  2.06 (1.39- 3.04) | 0.09  <0.001 |
| Histology  S＋EM  Others | 466  560 | 1  1.01 (0.86- 1.18) | 0.94 | 71  148 | 1  0.86 (0.50- 1.47) | 0.58 | 290  272 | 1  0.97 (0.78- 1.20) | 0.76 | 105  140 | 1  1.96 (1.49- 2.59) | <0.001 |
| Chemotherapy  Yes  No | 775  251 | 1  0.79 (0.65- 0.96) | 0.02 | 92  127 | 1  1.18 (0.70- 1.97) | 0.54 | 482  80 | 1  1.18 (0.88- 1.59) | 0.26 | 201  44 | 1  2.23 (1.63- 3.22) | <0.001 |
| Diagnostic period  2004-2007  2008-2011  2012-2015  2016-2018 | 185  240  330  271 | 1  0.91 (0.73- 1.14)  0.75 (0.61- 0.94)  0.72 (0.56- 0.92) | 0.42  0.01  0.01 | 49  43  69  58 | 1  0.74 (0.40- 1.37)  0.47 (0.24- 0.92)  0.25 (0.08- 0.75) | 0.33  0.03  0.01 | 90  132  184  156 | 1  0.77 (0.57- 1.04)  0.73 (0.54- 0.98)  0.68 (0.49- 0.96) | 0.09  0.04  0.03 | 46  65  77  57 | 1  1.05 (0.72- 1.55)  0.76 (0.52- 1.11)  0.87 (0.57- 1.33) | 0.79  0.16  0.53 |

HR, hazard ratio; CI, confidence interval; n, number of patients; Op (−), no-surgery group; S+EM,

serous adenocarcinoma/endometrioid adenocarcinoma.

Table S4. Univariate analysis of overall survival according to surgical outcome in patients aged ≥80 years.

|  | **All cases** | | | **Local disease** | | | **Regional disease** | | | **Distant disease** | | |
| --- | --- | --- | --- | --- | --- | --- | --- | --- | --- | --- | --- | --- |
|  | n | HR (95% CI) | P value | n | HR (95% CI) | P value | n | HR (95% CI) | P value | n | HR (95% CI) | P value |
| Disease extent  Local  Regional  Distant | 62  177  100 | 1  2.69 (1.81- 4.00)  5.74 (3.76- 8.75) | <0.001  <0.001 |  |  |  |  |  |  |  |  |  |
| Surgical outcome  No residual tumor  Residual macroscopic tumor  Op (−) | 124  71  144 | 1  2.48 (1.74- 3.52)  5.62 (4.14- 7.64) | <0.001  <0.001 | 54  2  6 | 1  0.48 (0.06- 3.64)  8.94 (3.14- 25.47) | 0.48  <0.001 | 57  49  71 | 1  2.13 (1.34- 3.40)  4.32 (2.80- 6.70) | 0.002  <0.001 | 13  20  67 | 1  1.53 (0.70- 3.32)  3.36 (1.71- 6.62) | 0.28  <0.001 |
| Histology  S＋EM  Others | 121  218 | 1  1.63 (1.25- 2.11) | <0.001 | 21  41 | 1  1.45 (0.63- 3.33) | 0.38 | 77  100 | 1  1.69 (1.20- 2.38) | 0.003 | 23  77 | 1  1.37 (0.85- 2.21) | 0.20 |
| Chemotherapy  Yes  No | 137  202 | 1  1.20 (0.94- 1.54) | 0.14 | 3  59 | 1  2.05 (0.28- 15.2) | 0.48 | 88  89 | 1  1.68 (1.20- 2.35) | 0.003 | 46  54 | 1  2.69 (1.76- 4.11) | <0.001 |
| Diagnostic period  2004-2007  2008-2011  2012-2015  2016-2018 | 45  73  109  112 | 1  0.98 (0.67- 1.45)  0.94 (0.65- 1.37)  0.82 (0.55- 1.22) | 0.93  0.74  0.33 | 13  13  15  21 | 1  0.72 (0.30- 1.77)  0.57 (0.20- 1.62)  0.61 (0.19- 1.90) | 0.48  0.29  0.39 | 16  39  64  58 | 1  1.15 (0.63- 2.11)  0.95 (0.53- 1.69)  0.92 (0.50- 1.69) | 0.65  0.86  0.80 | 16  21  30  33 | 1  1.03 (0.52- 20.3)  1.10 (0.59- 20.7)  0.72 (0.38- 1.36) | 0.93  0.76  0.31 |

HR, hazard ratio; CI, confidence interval; n, number of patients; Op (−), no-surgery group; S+EM,

serous adenocarcinoma/endometrioid adenocarcinoma.

Table S5

| Number of factors | **Op (+), n=995** |  | **Op (-), n=370** |  | **OR (95% CI)** | **P value** |
| --- | --- | --- | --- | --- | --- | --- |
|  | n | % | n | % |  |  |
| 0-2 | 608 | 82.7 | 127 | 17.3 | 1 |  |
| 3-5 | 387 | 61.4 | 243 | 38.6 | 3.00 (2.33- 3.89) | <0.001 |

OR, Odds ratio; CI, confidence interval; n, number of patients; Op (+), surgery group; Op (−), no-surgery group.
